# Exploring the challenges of RNAi-based strategies for crop protection

**DOI:** 10.1007/s44307-024-00031-x

**Published:** 2024-07-15

**Authors:** Jian-Hua Zhao, Qing-Yan Liu, Zong-Ming Xie, Hui-Shan Guo

**Affiliations:** 1 State Key Laboratory of Plant Genomics, Institute of Microbiology, Chinese Academy of Sciences, Beijing, China; 2 CAS Center for Excellence in Biotic Interactions, University of Chinese Academy of Sciences, Beijing, China; 3 Institute of Cotton Research, Xinjiang Academy of Agricultural and Reclamation Science, Shihezi, China

**Keywords:** RNAi, HIGS, SIGS, MIGS, RNAi inheritance

## Abstract

RNA silencing (or RNA interference, RNAi) initiated by double-stranded RNAs is a conserved mechanism for regulating gene expression in eukaryotes. RNAi-based crop protection strategies, including host-induced gene silencing (HIGS), spray-induced gene silencing (SIGS) and microbe-induced gene silencing (MIGS), have been successfully used against various pests and pathogens. Here, we highlight the challenges surrounding dsRNA design, large-scale production of dsRNA and dsRNA delivery systems. Addressing these questions will accelerate the lab-to-field transition of RNAi-based strategies. Moreover, based on studies of exogenous dsRNA-induced RNAi inheritance in *Caenorhabditis elegans*, we speculate that RNAi-based strategies would confer longer-lasting protection for crops against pests or fungal pathogens.

## Introduction

The increasing demand for food by the growing population, coupled with limited agricultural land availability, presents enormous challenges for modern agriculture (De Schutter et al. 2021; Hezakiel et al. 2023; Rank and Koch 2021). Pests and pathogens serve as the major biotic factors reducing cultivated land and crop yield worldwide (De Schutter et al. 2021; Dietz-Pfeilstetter et al. 2021; Rank and Koch 2021). To date, crop protection from these biological stresses mainly relies on the use of chemical pesticides. However, the improper use of chemical pesticides has led to environmental pollution and a loss of biodiversity, as well as the emergence of resistance in pest and pathogen populations (De Schutter et al. 2021; Rank and Koch 2021). To meet increasing food demands, chemical pesticide-free and safe alternatives are desired to facilitate sustainable agriculture. Disease resistance breeding, including cross-breeding, mutation breeding and transgenic breeding, is currently the main environmentally friendly strategy for crop improvement. Nonetheless, cross-breeding takes many years to introduce desirable alleles; mutation breeding is limited by screening large numbers of mutants; transgenic breeding requires the overexpression of exogenous resistance genes, which is restricted by a lack of resistance resources (Chen et al. 2019). Therefore, there is a pressing need for time-saving, convenient and independent resistant cultivar technology to protect crops against pests and pathogens.

RNA silencing (or RNA interference, RNAi) is an evolutionarily conserved and sequence-specific mechanism that regulates gene expression in most eukaryotic organisms (Chen and Rechavi 2022; Guo et al. 2019; Zhao and Guo 2022). In general, endogenous or exogenous double-stranded RNAs (dsRNAs) are processed into 18- to 30-nt small RNAs (sRNAs), including small interfering RNAs (siRNAs) and microRNAs (miRNAs). These sRNAs recognize complementary mRNAs or DNAs to regulate gene expression at either the transcriptional level or the posttranscriptional level by cleaving mRNA and inhibiting translation, DNA methylation and chromatin modification (Chen and Rechavi 2022; Guo et al. 2019; Zhao and Guo 2022). Due to the possibility and ease of designing dsRNA, RNAi-based strategies have been developed for crop protection.

Growing evidence shows successful applications of RNAi-based strategies for crop protection (Hou and Ma 2020; Liu et al. 2020; Zhao et al. 2021a; Zhu and Palli 2020). Even so, previous studies reported that dsRNAs are unable to induce gene silencing in *Ustilago maydis*, owing to the loss of the RNAi component proteins (Kamper et al. 2006; Keon et al. 1999; Laurie et al. 2008; Nakayashiki et al. 2006). Moreover, exogenous dsRNAs are not efficiently processed into sRNAs even in plant cells (Dalakouras et al. 2018; Uslu et al. 2020). A recent study attempted to engineer a bacterial symbiont of the insect to knock down the pea aphid gene by continuously supplying dsRNAs inside the insect body. However, they failed to achieve the expected phenotypic changes with either target (Elston et al. 2023). Therefore, the lab-to-field transition requires further development. Here, we highlight the challenges of dsRNA design, large-scale production of dsRNA and dsRNA delivery. Based on the discoveries of trans-kingdom RNAi and interspecies RNAi, we raise the fascinating question of transgenerational RNAi induced by exogenous RNA. Answering these questions will accelerate the lab-to-field transition of RNAi-based crop protection strategies.

## RNAi-based strategies for crop protection

Before the functional mechanisms of RNAi were clearly deciphered, the expression of homologous dsRNA was successfully applied to protect host plants against diverse viruses by initiating RNAi (Beachy et al. 1990; Guo and Garcia 1997; Lomonossoff 1995). For the past several decades, the strategy of expressing dsRNAs in plants to silence essential genes, termed host-induced gene silencing (HIGS), has been successful in enhancing resistance to pests or pathogens (Kierzek and Kierzek 2003; Nowara et al. 2010; Zhang et al. 2016a; Zhao and Guo 2022). Due to its independent of resistant cultivars, effectiveness, and environmental friendliness, HIGS technology is considered an ideal strategy for crop breeding (Hua et al. 2018; Zhao et al. 2021a). However, the broader application of HIGS is limited by a lack of transformation technology in many crop species. The alternative strategy of spray-induced gene silencing (SIGS) avoids crop transformation and initiated an era of RNAi-based biopesticides (Islam and Sherif 2020; Koch et al. 2016). This approach protects crops by spraying exogenous dsRNA or sRNA to silence target genes in pests and pathogens. dsRNA, as the active component, degrades rapidly in complex agricultural environments, avoiding unnecessary biosafety and environmental safety issues. Correspondingly, the instability of dsRNA in the environment increases the challenge of applying RNAi-based biopesticides (Luo et al. 2024). The successful application of SIGS for crop protection requires serious consideration of the application method and application rate. Numerous products have been developed to enhance the stability and persistence of dsRNA under field conditions. In one study, it was reported that dsRNAs loaded on layered double hydroxide can be detected on sprayed leaves even 30 days after application (Mitter et al. 2017). In another study, the detailed cellular process and mechanism of nanoparticle-mediated RNAi were visualized and elucidated (Ma et al. 2022). However, the impact of the stabilizing products on the environment and nontarget organisms needs to be considered (Bachman et al. 2020; De Schutter et al. 2021).

Recently, with the discovery of interspecies RNAi in rhizosphere fungi, an emerging alternative termed microbe-induced gene silencing (MIGS) has been exploited to protect various crops against distinct pathogenic microorganisms (Wen et al. 2023). In the MIGS scheme, an indigenous *Trichoderma harzianum* strain was selected as a chassis to produce dsRNAs that silence an attractive fungicide target (protein O-mannosyltransferase, PMT) against phytopathogens. Furthermore, abundant sRNAs generated from artificial dsRNAs were detected in the culture supernatants of engineered *T. harzianum*. It was demonstrated that the growth of *Verticillium dahliae* and *Fusarium oxysporum* was suppressed by two engineered *T. harzianum* strains inducing interspecies RNAi in a sequence-specific manner. Significantly, these engineered strains exhibited a stronger capacity than the chassis for plant protection (Wen et al. 2023). These results open up the possibility of improving agriculture with a low toxicity and environmentally friendly RNAi-based method (Fang 2024). In another study, *Beauveria bassiana,* which can infect host insects by directly penetrating the exoskeleton, was engineered to express immunosuppressive miRNAs in *Aedes*. During infection, these miRNAs generated from engineered *B. bassiana* suppress mosquito immunity and increase fungal virulence by silencing target genes, providing more effective and safer biocontrol of mosquitoes and other insect pests (Cui et al. 2022).

Due to the low toxicity, environmentally friendly and easy design of dsRNAs, RNAi-based strategies have drawn much attention from scientific research groups and market activities.

## The RNA structure is an important factor for dsRNA design

**Fig. 1:**
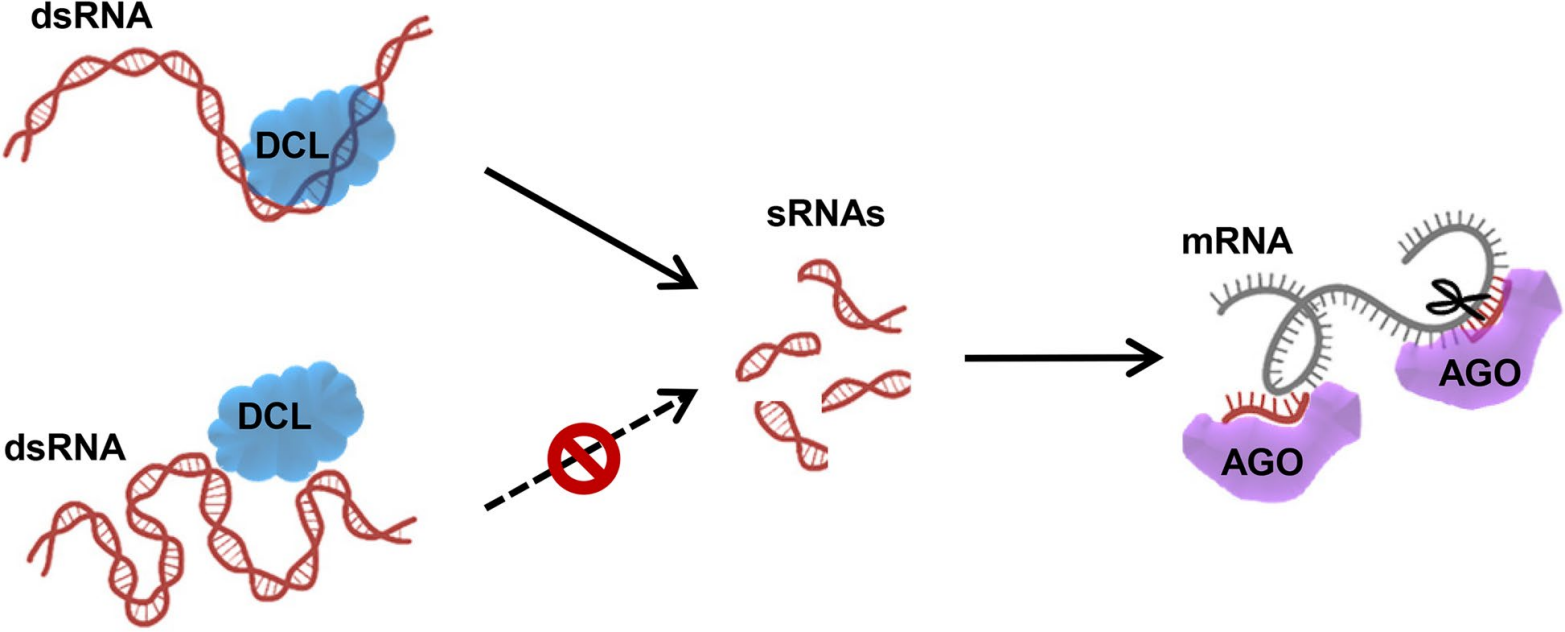
RNA structures affect the functions of RNAi key components including DCL and AGO proteins

To develop successful RNAi-based crop protection strategies, including HIGS, SIGS and MIGS, dsRNA design is of primary importance. Once an appropriate gene is selected for silencing, determining which region of the gene to target is critical for maximizing efficiency (Secic and Kogel 2021). In a previous study, several sRNAs were designed to target different regions of the same target mRNAs of human tissue factor (TF). Northern blot results showed that only a few of these sRNAs markedly inhibited TF expression (Holen et al. 2002). In plants, the protective efficacy of exogenous application of dsRNAs against tomato spotted wilt virus varies according to the targeted region (Tabein et al. 2020). To engineer effective RNA silencing and virus resistance in plants, the natural cleavage hotspots within the 3’ untranslated region (UTR) of the cucumber mosaic virus (CMV) genome were dissected. The artificial microRNAs (amiRNAs) that target putative sRNA accessible target sites confer high resistance to CMV (Duan et al. 2008). This study demonstrated the positional effect of sRNAs targeting mRNAs with a credible experimental approach. Overall, the efficiency of dsRNAs for targeting different regions on the same RNA is highly variable (Fig. 1). The great potential of RNAi-based crop protection strategies has evoked extensive interest in determining rules for improving dsRNA efficiency.

Target accessibility is the basic requirement for sRNA-mediated silencing (Duan et al. 2008; Gruber et al. 2023). Under natural conditions, however, RNA is always folded into a complex structure, resulting in inaccessibility to sRNA. A live-cell single-molecule imaging assay provided evidence for mRNA structure dynamics in the control of target recognition (Ruijtenberg et al. 2020). There is clear evidence that the tRNA-like structure within the 3’ UTR of CMV restricts amiRNA-mediated cleavage by impeding target access (Duan et al. 2008). Moreover, human immunodeficiency virus type 1 can escape RNAi-mediated inhibition by mutations that alter the target RNA secondary structure (Westerhout et al. 2005). Many studies have demonstrated that the mRNA structure at the targeted region is the main cause of the positional effect (Luo and Chang 2004). To date, several computational models have been used to design dsRNA by computing the accessibility of the target site based on mRNA structure (Table 1) (Chan et al. 2009; Heale et al. 2005; Kanoria et al. 2016; Luo and Chang 2004; Qureshi et al. 2013; Rennie et al. 2019; Schubert et al. 2005; Sciabola et al. 2013; Shao et al. 2007; Tafer et al. 2008). In addition to the target site, a previous study showed a strong inverse correlation between the degree of sRNA structure formation and gene silencing. They reported that unstructured siRNAs mediate the strongest silencing (Patzel et al. 2005).

**Table 1:** The representative computational models for dsRNA design based on mRNA structure

Names	Characteristics	References
H-b index	The gene-silencing effect is inversely dependent on the H-b index	Luo and Chang 2004
-	Combining secondary structure prediction method with duplex-end differential calculations	Heale et al. 2005
-	Systematic analysis of intentionally designed binding regions	Schubert et al. 2005
△G_disruption_	A quantitative measure of the structural accessibility at the target site	Shao et al. 2007
RNAxs	Combining known siRNA functionality criteria with target site accessibility	Tafer et al. 2008
-	Three-dimensional descriptors were used to improve the discrimination between active and inactive siRNAs in a statistical model	Sciabola et al. 2013
VIRsiRNApred	The first algorithm for predicting inhibition efficacy of viral siRNAs	Qureshi et al. 2013
STarMir	The model incorporated thermodynamic, structural, and sequence features	Kanoria et al. 2016; Rennie et al. 2019

In addition to sequence, diverse factors can modulate RNA structures (Nguyen et al. 2018). For example, *N*^6^-methyladenosine (m^6^A) can affect RNA folding stability (Hudson et al. 2013; Kierzek and Kierzek 2003; Kierzek et al. 2006, 2022; Roost et al. 2015; Wright et al. 2018). Moreover, m^6^A-dependent RNA structural switches regulate RNA‒protein interactions by altering protein accessibility (Lewis et al. 2017; Liu et al. 2015). We speculate that chemical modifications affect dsRNA processing or sRNA targeting by regulating the structure-dependent accessibility of RNAi pathway proteins (Fig. 1). Notably, various chemical modifications of miRNAs alter their targeting properties and physiological effects in different ways (Hwang et al. 2023).

Technological advances for probing RNA secondary structures are used to uncover the biological roles of RNA structures. Small-molecule modification-based methods, such as fragmentation sequencing (FragSeq) (Underwood et al. 2010) and parallel analysis of RNA structures (PARS) (Kertesz et al. 2010), can be used for transcriptome-wide RNA structure analysis via deep sequencing. Compared to small-molecule modification-based methods, crosslinking and proximity ligation-based methods can directly capture the RNA-RNA interactions (Wang et al. 2021). With the increasing availability of RNA structure data, computational approaches based on machine learning have been developed to predict RNA secondary structure (Zhao et al. 2021b). With the assistance of experimental and computational methods, efficient and specific dsRNAs are easier to design.

## Large-scale production of dsRNA

A key step in developing a successful RNAi-based crop protection strategy is to produce efficient amounts of dsRNA or sRNA. Unlike in HIGS and MIGS, host plants and engineered microorganisms used as bioreactors continuously generate dsRNA or sRNA, cost-effective dsRNA synthesis is needed to meet the requirement for the commercial application of RNAi-based biopesticides.

**Fig. 2:**
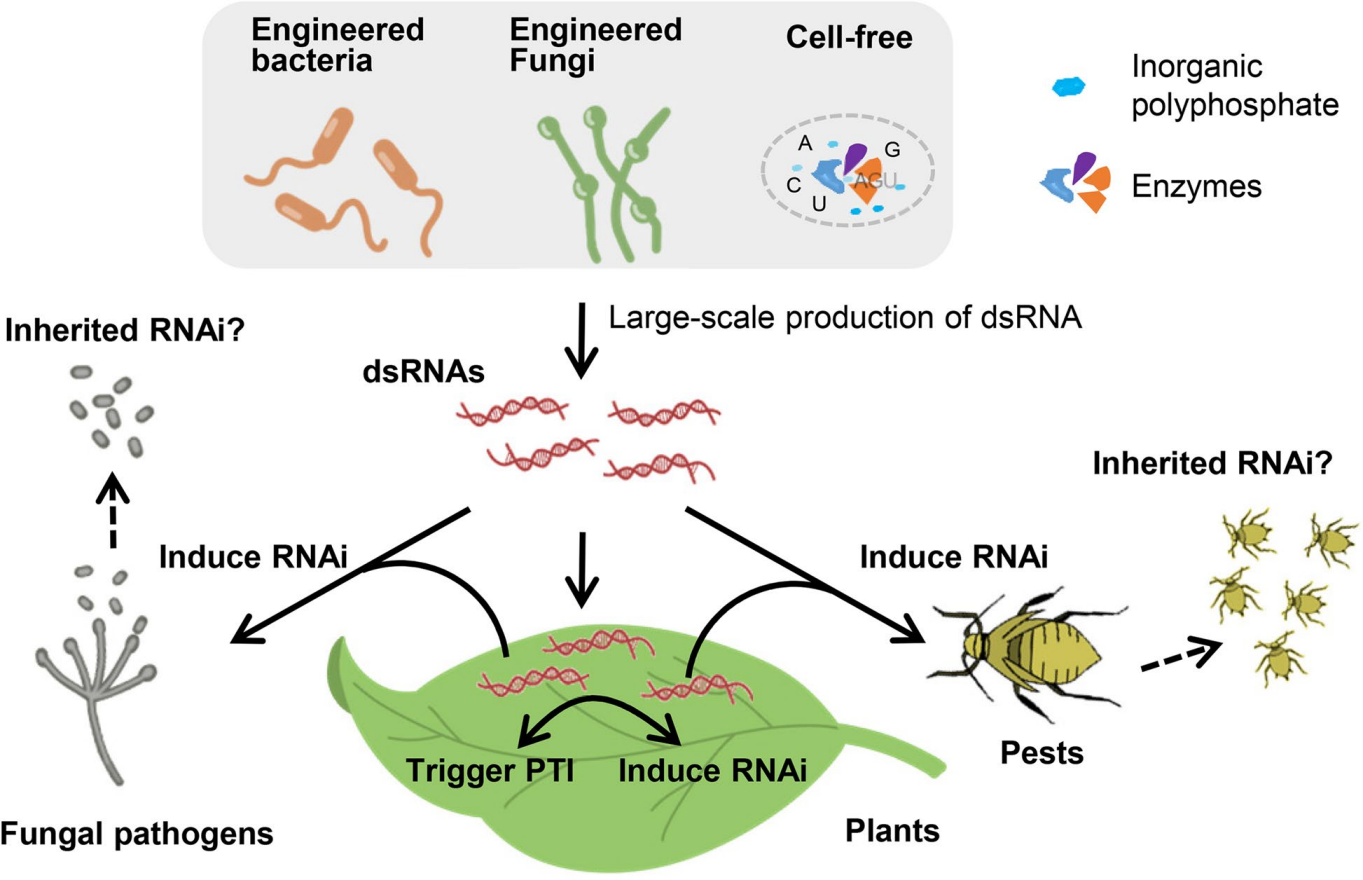
Exogenous dsRNAs behave in plants, fungal pathogens and pests

There is a lack of known RNAi mechanisms in prokaryotes. Interestingly, rhizobial transfer RNA (tRNA)-derived small RNA fragments modulate host genes associated with nodule initiation and development by hijacking the host RNAi machinery to enhance nodulation efficiency in legumes (Ren et al. 2019). In previous studies, crude extracts of bacterially expressed dsRNA were shown to protect different plants against various viruses (Gan et al. 2010; Tenllado et al. 2003; Yin et al. 2010). These results indicated that microbial fermentation potentially offers a cost-effective approach to manufacture dsRNA for commercial field applications (Fig. 2). The utilization of raw fermentation materials would further lower the cost of RNAi-based biopesticides. Compared to transcription in vitro, the RNA synthesis reaction cannot be directly controlled during fermentation, resulting in compromises in yield and purity (Rodrigues et al. 2021). In recent years, GreenLight Biosciences has developed a cell-free dsRNA production platform to yield high-quality dsRNA at minimal capital expenditure (Fig. 2). In the cell-free dsRNA production process, cellular RNA from inexpensive biomass, such as spent yeast material from industrial fermentation processes, is digested into nucleoside 5’-monophosphate monomers (NMPs) by a ribonuclease. These NMPs are converted to nucleoside triphosphates (NTPs) to replace highly purified NTPs in vitro transcription system. Inorganic polyphosphate, instead of exogenous adenosine triphosphate, is used as an energy source for RNA synthesis. The necessary enzymes are sourced from thermophilic organisms and produced in mesophilic organisms. Prior to RNA synthesis, simple heat treatment can mitigate the deleterious effects of decreased dsRNA production (Rodrigues et al. 2021). Using a cell-free platform, GreenLight produced the first sprayable dsRNA bioinsecticide targeting the *Leptinotarsa decemlineata* gene (Rodrigues et al. 2021).

Nevertheless, the results showing that dsRNA synthesized in vitro (Uslu et al. 2020) or generated in engineered bacteria (Elston et al. 2023) fails to induce silencing deserve attention. Two dsRNAs matching the green fluorescence protein (GFP)-encoding gene sequence were sprayed onto the leaves of *Nicotiana benthamiana* expressing GFP (16C) by a high-pressure spraying procedure. These dsRNAs did not induce silencing of the *GFP* reporter gene in 16C plants (Uslu et al. 2020). In another study, laboratory *Escherichia coli* and the native aphid symbiont *Serratia symbiotica* were engineered to produce dsRNAs targeting the salivary effector protein (C002) or ecdysone receptor (*EcR*) gene. qPCR results showed that dsRNAs were expressed by bacteria within the aphid body. However, the expected results of the aphid phenotype variation or knockdown of mRNA levels were not consistent (Elston et al. 2023). One possible explanation for these results is that dsRNAs synthesized in vitro or in bacteria cannot fold in proper structures, causing that they are not recognized by RNAi effectors.

In conclusion, dsRNA synthesis not only takes into consideration cost but also effectiveness. At present, fungal fermentation is likely the best choice for dsRNA synthesis.

## Delivery of dsRNA to targeted organisms

A critical requirement for RNAi-based crop protection against pathogens is the successful delivery of dsRNA. Although the mechanisms of sRNA transfer between donor and recipient organisms require further investigation, exogenous RNAs can successfully induce gene silencing in recipient organisms. The SIGS strategy faces challenges in the delivery of functional RNA to the target organism. Several classes of artificial nanomaterials have been developed to increase the stability of dsRNA in the environment (Luo et al. 2024; Rank and Koch 2021; Secic and Kogel 2021). However, how nanoformulated RNA biopesticides behave in unpredictable and highly dynamic field environments needs further investigation (Rank and Koch 2021).

### Nanocarrier-mediated dsRNA delivery

Growing evidence shows that nanomaterials can enhance SIGS efficiency, although there are limited data on the biosafety of nanomaterials, such as their influence on crop growth, soil biodiversity, nontarget organisms and human health (Lai 2015; Luo et al. 2024). A previous study investigated the effects of silver nanoparticles (AgNPs) on rice seed germination and seedling growth. The results revealed that AgNPs decreased the level of seed germination and subsequent growth (Thuesombat et al. 2014). Carbon dots, which exhibit very low toxicity and have been used for the transfection of RNA into animal and plant cells, provide an alternative to nanoparticles made from heavy metals (Cheon et al. 2017; Wang et al. 2014). A comprehensive cytotoxic study revealed principal differences in the toxicity of carbon dots at the cellular level. The authors found that carbon dots changed the phase of the cell cycle with or without entering the cell nucleus (Havrdova et al. 2016). The biotoxic effects of carbon-based nanomaterials on the microbial community have also been confirmed (Yang et al. 2021). The variation in the functional microbial community and the inhibition of enzyme activities caused a decrease in nitrogen removal efficiency in the soil, leading to a reduction in the diversity and abundance of specific microbial populations (Luo et al. 2024; Yang et al. 2021). These results strengthen our understanding of environmental safety information and attract attention to the environmental risk assessment of RNA biopesticides with nanomaterials as carriers.

### Natural systems for dsRNA delivery

In the pharmaceutical industry, several delivery vehicles have been developed to efficiently package and safely deliver therapeutic RNA cargoes to specific tissues (Madigan et al. 2024). However, intravenous delivery of dsRNAs or siRNAs encapsulated in liposomes but not naked RNAs stimulates the innate immune response in mice in a sequence-dependent manner (Judge et al. 2005). Recently, natural delivery systems from the human genome have been used to deliver cargo mRNAs to mammalian cells. In the human genome, homologs of the capsid protein (Gag) of long terminal repeat retrotransposons and retroviruses can form virus-like particles (VLPs), which are domesticated from integrating viruses and mobile genetic elements (Segel et al. 2021). One Gag homolog, PEG10, which preferentially binds and facilitates vesicular secretion of its own mRNA, was engineered to package, secrete, and deliver specific RNAs (Segel et al. 2021). *PNMA2*, which encodes a gag-like capsid domain, forms icosahedral capsids and traffics between cells but does not naturally encapsidate RNAs. Based on its cryoelectron microscopy structure, engineered PNMA2 particles were invested with RNA packaging ability and functioned as delivery vehicles in mammalian cell lines (Madigan et al. 2024). To improve dsRNA delivery and RNAi efficacy in lepidopteran insects, a fusion protein consisting of *Galanthus nivalis* agglutinin and a dsRNA binding domain (GNA:dsRBD) was used to design a lectin-based dsRNA delivery system. The results showed that GNA:dsRBD increases dsRNA uptake and transfection efficiency in lepidopteran midgut cells, resulting in efficient RNAi enhancement. Compared to naked dsRNA and GNA:dsRBD, GNA:dsRBD-dsRNA significantly increased insect mortality (Martinez et al. 2021). The Arabidopsis Dicer-like 3 (DCL3) protein is well known for its production of 24-nt sRNAs involved in RNA-induced DNA methylation. A recent study showed that DCL3 is involved in systemic RNA silencing through its RNA binding activity but not processing activity (Li et al. 2024). Although not enough is known about how the RBD domain of DCL3 accelerates systemic RNAi, there is tremendous potential for the use of the engineered RBD domain as a dsRNA delivery modality.

### Exogenous dsRNAs trigger host immunity response

Previous studies have shown that exogenous dsRNAs elicit antiviral defense (Huang et al. 2023). RNAi is the major mechanism by which plants defend against viruses. dsRNAs are generally associated with viral replication, which elicits host RNAi pathway-mediated degradation of the viral genome (Huang et al. 2023) (Fig. 2). Exogenous dsRNAs that share conserved molecular patterns with viral replicative intermediates can also be cleaved by DCL proteins. In addition, dsRNAs trigger a protein-mediated response known as pattern-triggered immunity (PTI) (Fig. 2). In Arabidopsis, both in vitro-generated dsRNAs and dsRNAs purified from virus-infected plants induced typical PTI responses, in which dsRNAs function as conserved microbe- or pathogen-associated molecular patterns. A signaling cascade involving SERK1 and a specific dsRNA receptor but not DCLs was triggered (Niehl et al. 2016). Unlike the bacterial and fungal elicitor-mediated PTI, a reactive oxygen species burst was undetectable. Interestingly, dsRNA-induced PTI restricted the progression of virus infection by triggering callose deposition at plasmodesmata, whereas viral movement proteins from different viruses suppressed the dsRNA-induced host response (Huang et al. 2023). Whether dsRNA-triggered plant immunity affects the function of biopesticides needs further study. In animal models, exogenous dsRNAs also stimulate the innate immune response in vitro systems that leverage transfection reagents and high RNA concentrations (Petrick et al. 2013; Rodrigues and Petrick 2020). Toll-like receptors, dsRNA binding protein kinases and RNA helicases play roles in the dsRNA-mediated innate immune response (Robbins et al. 2009). To exert biopesticide functions for pest control, dsRNA inevitably passes through the gut, where diverse microbial communities thrive. The ingestion of dsRNAs caused dysbiosis of the gut bacteria of the leaf beetle *Plagiodera versicolora*. The growth of the gut bacterium *Pseudomonas putida* is promoted by dsRNA degradation products. *P. putida* transitioned from a commensal lifestyle to a pathogenic lifestyle and accelerated the death of *P. versicolora* (Xu et al. 2021).

Based on the above discussion, the examination of target gene expression is necessary. Although dsRNAs function in a sequence-specific manner, their toxicity to nontarget organisms cannot be ignored. The biosafety of nanomaterials as delivery vehicles should be assessed independently of dsRNAs.

## Exogenous RNA-mediated inherited silencing

*Caenorhabditis elegans* has emerged as one of the leading model organisms for studying intergenerational RNAi induced by heritable sRNAs (Frolows and Ashe 2021). sRNAs can repress gene expression by mediating specific histone modifications in the nucleus. Heritable sRNAs are detectable in the progeny of *C. elegans* before the appearance of histone modifications, indicating that heritable sRNAs are sufficient for intergenerational RNAi (Burton et al. 2011). Using a fluorescently labeled dsRNA, another study demonstrated that exogenous dsRNAs ingested by *C. elegans* were directly transferred into progeny without entry into the cytosol. These dsRNAs were imported from the extracellular space into oocytes along with yolk by RME2-mediated endocytosis. Then, dsRNAs reach the cytosol of embryos and spread between cells to silence the target gene in progeny (Marre et al. 2016). The inheritance of silencing usually diminishes drastically after 3–5 generations, which was dubbed “the bottleneck to RNAi inheritance” (Alcazar et al. 2008; Houri-Zeevi and Rechavi 2017). These results raise the question of whether inherited dsRNA-induced silencing becomes diluted over several generations. Subsequent studies demonstrated that a regulated but passive dilution mechanism controls exogenous dsRNA-induced RNAi inheritance. Surprisingly, long RNAi responses lasting over 80 generations, which depend upon the activity of genes involved in chromatin remodeling, were detected in *C. elegans* (Vastenhouw et al. 2006). One study proposed that a mechanism might enable RNAi inheritance in the parental generation but actively terminate it in the progeny (Houri-Ze'evi et al. 2016). The authors examined whether the activity of the RNAi system affects the duration of heritable silencing. In this study, feeding GFP-labeled *C. elegans* to bacteria that produce anti-GFP dsRNA induced silencing in the progeny. Consistent with previous reports, the inhibition of GFP expression could not be detected after approximately four generations. As a parallel test, the progeny were transferred to plates with bacteria expressing different dsRNAs (such as anti-mCherry dsRNA) that cannot silence the *GFP* gene (second dsRNA triggers). Compared to the progeny that were exposed to bacteria expressing the empty vector, the progeny that were exposed to bacteria expressing the second dsRNA trigger exhibited much stronger inherited GFP silencing (Houri-Ze'evi et al. 2016). These results indicated that passive dilution is likely not the main factor that diminishes inherited RNAi.

Compared to chemical pesticides, RNAi-based strategies confer longer-lasting protection against pests or fungal pathogens due to inherited RNAi. During *Verticillium dahliae* infection, cotton plants export miR159 and miR166 to inhibit virulence gene expression in fungal pathogens (Zhang et al. 2016b). The plant miRNAs were still detectable in fungi after 20 days of in vitro culture, implying that exogenous miRNAs are transported from one generation to the next in *V. dahliae*. It is worth examining whether sprayed dsRNAs and dsRNAs generated from engineered microbes can induce the inheritance of RNAi in pathogens (Fig. 2).

## Conclusions and perspectives

Many studies have shown that RNAi-based strategies, such as HIGS, SIGS and MIGS, successfully protect crops against pests and fungal pathogens. However, we still lack reliable results from field trials. To accelerate the lab-to-field transition of RNAi-based strategies, some questions surrounding dsRNA design, large-scale production of dsRNA and dsRNA delivery systems remain unanswered.

At present, there are no absolute rules for improving dsRNA efficiency. For dsRNA design, the three-dimensional structures of RNA molecules that are critical to their function should be considered. Despite decades of intense effort, few RNA structures are known, and predicting the structure of RNAs remains a great challenge (Townshend et al. 2021). With the development of structural biology and artificial intelligence, structure-guided dsRNA design offers the potential for new avenues in RNAi-based crop protection strategies. Microbial fermentation and cell-free platforms potentially offer a cost-effective approach to produce dsRNA for commercial field applications. However, how dsRNAs function in recipient organisms should be examined carefully. Inhibition of pathogenicity should be distinguished between RNAi-mediated and other pathway-induced effects, including the activity of the immune response and dysbiosis of the microbial community.

## Data Availability

Not applicable.
